# Late and Chronic Antibody-Mediated Rejection: Main Barrier to Long Term Graft Survival

**DOI:** 10.1155/2013/859761

**Published:** 2013-10-08

**Authors:** Qiquan Sun, Yang Yang

**Affiliations:** ^1^Department of Renal Transplantation, The Third Affiliated Hospital, Sun Yat-Sen University, 600 Tianhe Road, Guangzhou 510630, China; ^2^Department of Liver Transplantation, The Third Affiliated Hospital, Sun Yat-Sen University, 600 Tianhe Road, Guangzhou 510630, China

## Abstract

Antibody-mediated rejection (AMR) is an important cause of graft loss after organ transplantation. It is caused by anti-donor-specific antibodies especially anti-HLA antibodies. C4d had been regarded as a diagnosis marker for AMR. Although most early AMR episodes can be successfully controlled or reversed, late and chronic AMR remains the leading cause of late graft loss. The strategies which work in early AMR have limited effect on late/chronic episodes. Here, we reviewed the lines of evidence that late/chronic AMR is the leading cause of late graft loss, characteristics of late AMR, and current strategies in managing late/chronic AMR. More effort should be put on the management of late/chronic AMR to make a better long term graft survival.

## 1. Introduction

Organ transplantation now represents the treatment of choice for patients developing end stage organ failure [1]. However, despite the now routine nature of clinical transplantation, even well-matched transplants are recognized and eventually destroyed by the host immune system [2]. The emerging of a new immunosuppressant has decreased the incidence of early graft loss, and even T-cell-mediated rejection occurs; it is usually easily controlled. However, the long term graft survival remains to be improved [3]. Although it was formerly held that alloreactive T cells are solely responsible for graft injury, it is now well recognized that antidonor alloantibodies are also an important barrier to long term graft survival [4, 5]. More and more lines of evidence suggest that antibody-mediated rejection (AMR) is predominance cause of late term graft loss [6, 7], especially late occurring AMR and chronic AMR (CAMR). Thus, strategies targeting alloantibody reactivity will be helpful in prolonging long term graft survival.

## 2. Antibody-Mediated Rejection

AMR is caused by anti-donor-specific antibodies, mostly anti-HLA antibodies [8, 9]. Some non-HLA antibodies also have been reported to induce AMR in rare cases. The phenotype of AMR ranges from hyperacute rejection, acute AMR, and chronic AMR. The diagnosis of AMR depends on typical histological lesions, C4d staining, and serum DSA detection. C4d, a protein from the classical complement activation cascade that remains attached to the site of complement activation, is regarded as a diagnosis marker for AMR. The introduction of C4d as marker of AMR aroused an ever-increasing interest in recognizing mechanisms of allograft rejection. However, C4d has several limitations in the diagnosis of AMR. For instance, it can be found in the majority of grafts with stable function in ABO-incompatible transplantations. On the other hand, a group of C4d-negative AMR has been recognized based on endothelial gene expression [10, 11]. About 40% of patients with endothelial-associated transcripts expression and chronic AMR features demonstrated no C4d staining. Similarly, C4d staining is only positive in about half of patients with transplant glomerulopathy [12, 13], which is a special form of chronic AMR. C4d-positive and -negative AMR share similar degrees of glomerulitis and peritubular capillaritis, similar frequencies of concurrent cell-mediated rejection, and both may occur early or late after transplantation, thus needing to be treated equally [14].

Obviously, a new marker for AMR is extremely necessary. It is reported that microcirculating inflammation is strongly correlated with alloantibody reactivity; however, whether it is can be used as maker of AMR is still of contradictory [15]. T-box expressed in T cells (T-bet), transcription factor for Th1, has been reported to be correlated with microcirculating inflammation both in acute and chronic AMR [16, 17], and the predominance of T-bet over GATA3 (transcription factor for Th2) is strongly correlated with AMR [16]. However, whether the ratio of T-bet/GATA3 can be used as a diagnosis maker for AMR needs further investigation.

### 2.1. Late/Chronic AMR

The importance of CAMR is increasingly recognized. It has been known as a major cause of late graft dysfunction in renal transplantation. Banff 07 consensus conference [18] described that the characteristics of chronic AMR were C4d deposition in the capillary basement membrane, the presence of circulating anti-donor antibodies, and morphologic evidence of chronic tissue injury such as glomerular double contours compatible with transplant glomerulopathy, peritubular capillary basement membrane multilayering, interstitial fibrosis/tubular atrophy, and fibrous arterial intimal thickening. Late occurring AMR may manifest as CAMR; however, according to Banff 07 meeting, the term “chronic” is not related to a certain time after transplantation but indicates morphological changes of remodeling seen in the allograft due to antibody-mediated injury [18], for example, double contours of glomerular basement membranes. Thus, it is not strange that late AMR can be acute AMR. However, both CAMR and late AMR have poor response to regular anti-AMR treatment, and they are sometimes discussed together.

### 2.2. Late AMR, a Special Clinical Entity?

AMR episodes occurring at different time periods seem to be different clinical subentities [19–21]. They have different risk factors, different clinical manifestations, and different outcomes (Table 1). Early AMR are usually correlated with sensitization, pre-existing alloantibodies, and rapid graft dysfunction and are usually easy to be controlled; while late AMR mostly correlated with withdrawal or reduction of immunosuppressants, noncompliance with immunosuppressive therapy. There is a relatively slow but progressive graft dysfunction; some patients have anemia and hypoalbuminemia. Late AMR have little response to antirejection strategies and thus correlate with poor graft outcomes [19, 20]. The significantly poorer outcome of late AMR is also observed in simultaneous pancreas-kidney transplantation [22], even under combined treatment of steroids, intravenous immunoglobuin (IVIG), and rituximab.

### 2.3. Late/Chronic AMR, Main Cause to Late Graft Loss

A eliminated term, chronic allograft nephropathy, had been regarded as a main cause of late graft loss [23]. However, chronic allograft nephropathy is actually like a can, which includs both immunological and nonimmunological parameters caused graft damage; thus, this term has been eliminated in Banff 2005 meeting [24]. Recent studies revealed that AMR is the leading cause of late grafts loss. In 2009, researchers from Dr. Holloran's group in Edmonton studied the phenotype of late kidney graft failure [6]; they found that patients with late index biopsies (>1 year) frequently displayed donor-specific HLA antibody (particularly class II) and microcirculation changes, including glomerulitis, glomerulopathy, capillaritis, capillary multilayering, and C4d staining. T-cell-mediated rejection rarely leads to graft failure. However, they found that 63% of late kidney failures after biopsy were attributable to AMR.

Further prospective study from the same group [7] investigated kidney transplants that progressed to failure after a biopsy for clinical indications. Similarly, they found that graft failure was rare after T-cell-mediated rejection and acute kidney injury while was common after AMR or glomerulonephritis. The majority of graft loss had evidence of AMR by the time of failure. Interestingly, pure T-cell-mediated rejection, acute kidney injury, and drug toxicity were not causes of loss. These findings are interesting and, however, are not strange as they are consistant with an early study which reported that all chronic rejection failures of kidney transplants preceded by development of HLA antibodies [25]. Recent study from Terasaki Foundation Laboratory revealed that 11% of the patients without detectable DSA at transplantation will have detectable DSA at 1 year; and over the next 4 years, the incidence of de novo DSA will increase to 20%. After de novo DSA development, 24% of the patients will fail within 3 years [26]. Given these findings, de novo DSA, especially anti-HLA class II antibodies [27], have to be considered as a primary risk factor for late allograft loss.

### 2.4. Natural History of Chronic AMR

The development of chronic AMR, especially TG usually takes years, and there still lacks of a perfect animal model to mimic the lesions during chronic AMR; thus, the natural history of chronic AMR is still not clear. Recently Wiebe et al. [28] have monitored a group of renal allograft recipients with de novo DSA, they proposed that posttransplant de novo DSA is probably preceded by an antibody-free period. Then, inflammatory events such as cellular rejection or graft infection might upregulate HLA expression on endothelial cells and stimulate B-cell allorecognition and subsequent long-lived plasma cells producing de novo DSA. De novo DSA binding vascular endothelium could induce injury through the activation of complement or recruitment of neutrophils, macrophage, or natural killer cells. Sustained microvascular inflammation eventually leads to progressive tissue damage and graft dysfunction. Cellular inflammation is often concomitant of AMR [29, 30] in either its acute or chronic form. However, the pathogenesis remains to be determined.

### 2.5. Antibody-Mediated Vascular Rejection

A recent population based study [31] revealed a new type of kidney rejection not presently included in classifications, which is an antibody-mediated vascular rejection. This study included 302 cases of acute biopsy-proven rejection. Four distinct patterns of kidney allograft rejection were identified, including T-cell-mediated vascular rejection (26 patients (9%)), antibodymediated vascular rejection (64 (21%)), T-cell-mediated rejection without vasculitis (139 (46%)), and AMR without vasculitis (73 (24%)). The graft survival is very poor in antibody-mediated vascular rejection, which has a 9 times higher graft loss incidence compared with T-cell-mediated rejection without vasculitis. Unfortunately, the authors did not provide how many of the AMR episodes were late AMR.

## 3. Management of Late/Chronic AMR

### 3.1. An Ounce of Prevention Is Worth a Pound of Cure

Chronic AMR is a B-cell-mediated production of immunoglobulin (Ig) G antibody against a transplanted organ. Based on this pathophysiologic condition, rituximab, IVIG, and bortezomib have been used as treatment for chronic AMR recently. However, till now, there is no standardized treatment for late/chronic AMR. The strategies that can effectively reverse early AMR do not work as well in late episodes; thus, an ounce of prevention is really worth a pound of cure [32]. As late AMR usually is caused by de novo DSA, posttransplant HLA alloantibody monitoring is of great importance for the prevention of antibody-mediated allograft injury [33]. Prevention of nonadherence and insufficient immunosuppression are additional important issues in the prevention of antibody-mediated allograft injury, as these factors are risk factor for late AMR. A recent study based on ABO-incompatible renal transplantation revealed that B-cell depletion protocols, such as splenectomy or rituximab administration, could reduce chronic AMR after kidney transplantation. Finally, the triple immunosuppressants protocol including mycophenolic acid, tacrolimus, and steroid can control antidonor antibody production in renal allograft recipients with chronic rejection [34] and seem to be superior to others in treating AMR [35, 36]; however, whether it can prevent the development of late AMR is not clear, see Table 2.

### 3.2. Rituximab/IVIG

Several single center studies showed that the combination treatment with rituximab/IVIG may be a useful strategy for the treatment of chronic AMR. In 2008, a German group [49] published their pilot study in six pediatric renal transplant recipients with chronic AMR. Their treatment regimen was four weekly doses of IVIG (1 g/kg body weight per dose), followed by a single dose of rituximab (375 mg/m^2^ body surface area) 1 week after the last IVIG infusion. Four of the six patients had good response to this treatment; they had improved or stabilized eGFR. Further prospective studies from the same group showed that under this treatment, in the means of eGFR, 70% patients responded to treatment as measured 6 months after intervention, and this response persisted over a 24-month observation period. The rationale for the rituximab/IVIG treatment was to use IVIG for its immunomodulatory action and then rituximab for prevention of further antibody production.

At the year 2009, Fehr et al. [50] published his work of using rituximab/IVIG treatment on four adult patients with chronic AMR. The result showed that rituximab/IVIG treatment improved kidney allograft function in all four patients, and donor-specific antibodies were reduced in 2 of 4 patients. The treatment regimen of this study was that on diagnosis of chronic AMR, all patients received intravenous steroid pulses (500–1000 mg once daily for 3 to 5 days) and rituximab (375 mg/m^2^ once on day 1), whereas IVIG (0.4 g/kg once daily on day 2 to 5) was given only to 3 patients. About the treatment safety, 3 out of 4 patients underwent therapy with rituximab/IVIG without side effects. One patient had severe, possibly rituximab-associated lung toxicity. Their study showed that rituximab/IVIG may be a useful strategy for the treatment of chronic AMR. Another pilot study showed that rituximab/IVIG treatment took effect in 3 out of 4 patients. Early stage of chronic AMR has better response than advanced stage [51]. Anyway, although rituximab/IVIG treatment takes effects in some CAMR cases, it is far from comparable to early AMR cases [22]. A retrospective study from Massachusetts General Hospital [52] studied the effect of rituximab followed by standard maintenance immunosuppression, they found that this protocol shows a therapeutic effect in 8 out of 14 CAMR. Response to rituximab was defined as decline or stabilization of serum creatinine for at least one year in this study.

### 3.3. Bortezomib

Bortezomib is a proteosome inhibitor that leads in vitro to apoptosis of alloantibody-producing plasma cells [53]. It has shown promising effect in early AMR cases [43]. Early reports of bortezomib-based AMR treatment demonstrated the ability of bortezomib to deplete plasma cells producing DSA, reduce DSA levels, provide histological improvement or resolution, and improve renal allograft function [54]. Initial results from a multicenter study showed [55] that bortezomib-based regimen reversed AMR in adult kidney, kidney/pancreas, and pediatric heart transplant recipients; a common bortezomib-based regimen demonstrated substantial DSA reductions, with more than half of the patients achieving a 45.0% reduction in DSA level. However, plasmapheresis has been performed every third day immediately before bortezomib therapy. In a chronic AMR rat cardiac transplant model, administration of bortezomib 60 or 80 days after transplantation may reduce antidonor MHC classes I and II Abs. Histological improvements were also observed with bortezomib administration, including reduction in C4d expression, interstitial fibrosis, and vasculopathy [56]. Unfortunately, it is not as effective in late AMR cases. Walsh et al. treated 30 episodes of AMR, and they found that early AMR patients demonstrated greater reduction in DSA and histologic resolution/improvement. They concluded that early and late AMR exhibit distinct immunologic characteristics and respond differently to proteosome inhibitor therapy.

### 3.4. Eculizumab

As complement plays an important role in the pathogenesis of AMR, complement-blocking agents could be used for the treatment of AMR. Eculizumab is a humanized monoclonal antibody against complement C5. It can bind to the C5 protein and inhibit conversion of C5 to C5b, thus preventing formation of the membrane attack complex (C5b–9). Eculizumab has been used to rescue atypical hemolytic uremic syndrome after renal transplantation [57]. A prospective study showed that eculizumab can reduce the incidence of AMR and transplant glomerulopathy in highly sensitized individuals when administered immediately after transplant [58]. Cases had been reported that eculizumab reverse AMR is associated with thrombotic microangiopathy [59]; it can even reverse severe AMR episode refractory to salvage splenectomy and daily plasmapheresis in ABO incompatible (ABOI) living donor kidney transplantation [45]. However, there is no evidence that eculizumab can be used for late AMR, and clinical trials are necessary to determine the optimal use of C5 inhibition.

### 3.5. Splenectomy

The spleen acts as a repository for memory B cells and plasma cells; thus, splenectomy is supposed to be effective in treating AMR. There is data suggesting that splenectomy alone can lead to rapid diuresis and immediate restoration of renal function [47]. Rescue splenectomy is currently regarded as last salvage option for AMR. There is a case that reported [60] that splenectomy is effective for treatment of CAMR after renal transplantation. However, clinical trials are needed to prove this finding.

## 4. AMR in Liver Transplantation

The liver allograft is generally regarded as relatively resistant to AMR. The resistance is attributed to a variety of characteristic features of liver which contribute to the clearing and dilution of antibodies or antigen-antibody complexes, such as Kupffer cell phagocytosis, large sinusoidal surface area, dual afferent hepatic blood supply, and secretion of soluble MHC class I antigen [61]. For many years, hyperacute rejection was thought not to occur, even when the ABO incompatible graft was used. However, subsequent increasing studies have shown that liver transplantation across the ABO blood type barrier (ABOi) is prone to AMR, which often leads to a poor clinical outcome. Unlike a reliable tissue marker of AMR in renal and cardiac allografts, the diagnostic utility and functional significance of C4d immunostaining in the liver allograft are controversial and less clearly formed. There are reports that showed that extensive C4d deposition is associated with AMR and correlated with graft survival. However, C4d deposition in liver was also detected in several other conditions, such as acute cellular rejection, chronic rejection, and recurrent diseases including hepatitis B, hepatitis C, and autoimmune hepatitis, and even preservation injury [62–64]. Therefore, the diagnosis of AMR in liver cannot be made on the basis of histological finding alone and requires other supportive features as well as the exclusion of other causes of graft dysfunction that can mimic the pathological changes occurring in AMR. However, the presence of diffuse C4d immunostaining (involving endothelium or stroma in >50% of portal tracts or sinusoids) provides supportive evidence for a diagnosis of AMR. Similar to renal transplantation, conventional T-cell-based immunosuppression usually seems less effective for cases with strictly defined AMR. Treatment with aggressive B-cell directed immunosuppression, including IVIG, plasmapheresis, and rituximab, is recommended to be used [61]. In animal models, antibody-mediated responses might play important roles in the development of chronic liver allograft rejection. However, the role of AMR in late liver graft loss is still underdetermined.

## 5. AMR in Other Organ Transplantation

AMR is also involved in other organ transplantation, especially for heart transplantation. DSA binding to the heart allograft causes myocardial injury predominantly through immune complex activation of the classical pathway of the complement cascade [65], and thus is a significant risk for allograft failure, cardiac allograft vasculopathy, and poor survival. C4d is accepted as a marker for AMR in cardiac allografts. The diagnosis of AMR has evolved from a clinical diagnosis to a primarily pathologic diagnosis based on histopathology and immunopathology. The ISHLT 2005 Working Formulation [66] recommended that AMR be diagnosed on the basis of (1) evaluation of the routinely processed and stained paraffin sections for endothelial-cell swelling and accumulations of intravascular macrophages; (2) immunophenotypic evidence of immunoglobulin (IgG, IgM, and/or IgA) and complement (C3d, C4d and/or C1q) deposition in capillaries by immunofluorescence (IF) on frozen sections and/or CD68 staining of intravascular macrophages in capillaries and C4d staining of capillaries by paraffin immunohistochemistry (IC). The final clinical diagnosis of AMR required evidence of allograft dysfunction and circulating donor-specific antibodies together with the histopathologic and immunophenotypic findings. Therapies include plasmapheresis, immunoadsorption columns, intravenous immune globulin, rituximab, and bortezomib. The combinations of steroid, IVIG, and plasmapheresis are suggested as initial therapies [67]. Late cardiac AMR caused by de novo DSA is also a serious problem; despite treatment consistent with current best practice, 46% of patients developed persistent cardiac dysfunction and their medium-term survival was poor [68].

Besides renal and heart transplantation, AMR is also a major complication causing graft injury after lung [69], pancreas [70], and intestinal [71] transplantation. Similarly, all the AMR are caused by DSA, and C4d is accepted as diagnosis marker. Antibody removal strategies are also used for these episodes. More studies are needed to understand these terms and improve their outcomes.

## Figures and Tables

**Table 1:** Early versus late AMR in renal transplant recipients.

	Early AMR	Late AMR
Main risk factor	Positive panel reactivity antibody before transplantation, including factors causing sensitization	Withdrawal or reduction of immunosuppressants Noncompliance with immunosuppressive therapy, young age
Antibody	Mostly pre-existing donor-specific antibodies	Mostly de novo donor-specific antibodies, especially HLA class-II antibodies
Clinical features	Very rapid graft dysfunction, significantly decreased urine output, and rapid graft dysfunction	Proteinuria, hypertension, progressive functional deterioration, and overt graft failure
Histology	ATN-like minimal inflammation; capillary and or glomerular inflammation and/or thrombosis; arterial—v3	May have chronic tissue injury, such as glomerular double contours, peritubular capillary basement membrane multilayering, interstitial fibrosis/tubular atrophy, and/or fibrous intimal thickening in arteries
Outcome	Good, mostly reversible	Usually poor

**Table 2:** Strategies to treat AMR.

Strategies	Mechanisms
Plasmapheresis [37], immunoadsorption [38]	Removal of donor-specific antibodies
IVIG [39, 40]	Multiple mechanisms, basically pleiotropic immunomodulation
Rituximab [41, 42]	Chimeric anti-CD 20 monoclonal antibody, depleting B cells
Bortezomib [43, 44]	Proteasome inhibitor, may cause apoptosis of normal plasma cells which in turn decreases alloantibody production
Eculizumab [45, 46]	Humanized monoclonal antibody anti-C5
Mycophenolic acid, tacrolimus [34, 35]	Inhibit production of DSA
Splenectomy [47, 48]	Immediate reduction of the B-cell and plasma cell pool

## Abbreviations

AMR: Antibody-mediated rejection
CAMR: Chronic antibody-mediated rejection
DSA: Donor-specific antibody
HLA: Human lymphocyte antigen
IVIG: Intravenous immunoglobuin.
